# Traumatic Brain Injury Screening and the Unmet Health Needs of Shelter-Seeking Women with Head Injuries Related to Intimate Partner Violence

**DOI:** 10.1089/whr.2021.0056

**Published:** 2021-12-07

**Authors:** Linda Denise Oakley, Jeneile Luebke, Natalie C. Dosch, Traci R. Snedden, Hector Hernadez, Melissa Lemke, Rick P. Voland

**Affiliations:** ^1^ School of Nursing, University of Wisconsin-Madison, Madison, Wisconsin, USA.; ^2^ School of Medicine, University of North Carolina at Chapel Hill, Chapel Hill, North Carolina, USA.; ^3^ Sojourner Family Peace Center, Milwaukee, Wisconsin, USA.; ^4^ Urban Medicine and Public Health Triumph Program, University of Wisconsin-Madison, Madison, Wisconsin, USA.

**Keywords:** IPV, survivors, TBI, Women's Shelters

## Abstract

***Background:*** Unmet health needs of women with head injuries sustained by intimate partner violence (IPV) include risk of traumatic brain injury (TBI). The purpose of this evaluation was to explore the potential effectiveness of TBI screening as a health promotion strategy for shelter-seeking women with IPV head injuries. We wanted to learn if shelter-seeking women, willing to disclose IPV, would accept TBI screening if offered.

***Methods:*** An extended version of the HELPS TBI screening tool and survey of daily symptoms and health needs were used to screen new residents of an urban shelter for women.

***Results:*** The participants (*N* = 18) primarily were educated black women with one or more self-reported IPV-related head injury. Most participants (77.8%) had positive TBI screens for probable brain injury. The majority (88.8%) lived with one or more daily symptoms they did not have before sustaining a IPV head injury. The symptoms reported most frequently were depression (88.9%), anxiety (77.8%), and headache (66.7%). All participants had one or more unmet health need. Although most (77.8%) needed to see a primary care provider, mental health care was the most important health need identified.

***Conclusions:*** TBI screening could be considered an effective health promotion strategy for IPV survivors if screening facilitates treatment for positive screens and other unmet health needs. Further research is needed to properly assess this.

## Background and Significance

Intimate partner violence (IPV), defined as physical violence, sexual violence, stalking, or psychological aggression by a current or former intimate partner, is a pervasive underrecognized public health problem with an estimated U.S. annual economic burden of $3.6 trillion.^1,2^ One in three U.S. women will experience IPV in her lifetime; one in five will survive the intense physical violence of being punched with a closed fist, beaten, strangled, or assaulted with a knife or gun.^3^ Because physically violent partners target the woman's head, neck, and face, U.S. emergency departments must document any unwitnessed head, neck, or facial injury as a sign of IPV.^4,5^

The frequency of singular IPV-related head and face injuries, not counting repeat head injuries in a violent relationship, for U.S. women is estimated to be as high as 92%.^6,7^ Consequently, researchers estimate that up to 75% of women survivors of IPV can have unrecognized traumatic brain injuries (TBI) and multiple health problems.^8^ This elevated risk of poor health requires that service providers and treating clinicians understand the uniqueness of IPV-related head injuries.

Because the risk of IPV, injury, or death peaks when women try to leave, find help, or report the violence,^8^ the potential health risks of unrecognized TBI in women with IPV head injuries seeking shelter must be assessed in all age groups.^9,10^ Compared with survivors of IPV with no head injuries, those who develop IPV-related TBI experience memory loss, hearing or vision problems, seizures, and vague difficulties with mental concentration.^11^ In addition to chronic neurological problems, including recurrent or persistent headaches,^7^ IPV-related head injuries also are associated with clinical psychiatric problems ranging from overwhelming distress, depression, and anxiety to potentially disabling posttraumatic stress disorder (PTSD).^12,13^

Unrecognized loss of cognitive functioning is a uniquely troubling health outcome of IPV-related head injuries. Alterations in cognitive functioning can silently increase the difficulty of leaving a violent relationship by limiting the women's ability to work and live independently.^13,14^ Those who remain in a violent relationship may become trapped in an ongoing cycle of violence and complications, including repeated head injuries, making the thought of leaving impossible to contemplate.^14,15^ When these IPV-related complications are compounded by common symptoms of IPV-related head injuries, such as headaches, memory problems, and depression, the risk of common chronic health problems, such as hypertension, also increases.^15^

Despite these well-documented health needs, few women who report an IPV-related head injury receive TBI screening. Recent findings suggest that the actual proportion of women with documented IPV-related head injuries who receive medical care at the time of their injury is about 21%.^7^ Women with a history of IPV also may choose not to put themselves in a situation where they are likely to be shamed and blamed for their circumstances, subjected to implicit bias, face exhausting systemic health care inequities, or more dangerous forms of IPV.^16^ Those seemingly able to face these challenges might be stopped by a far more overwhelming fear. Having lived with head injuries, they may have reason to fear being diagnosed with a condition that could put them in jeopardy.^6^

Based on our review of the literature, the essential health needs of women with IPV-related head injuries are screening to determine the probability of a TBI and assessment to identify related personal health needs. Experts suggest that emergency shelters, as first-responders, can also offer screenings and assessments.^7,17,18^ Given that shelters who screen residents for TBI could find that half, up to two-thirds, of the screens are positive for probable TBI, routine screening services must have an evidence-based protocol for positive TBI screens.^13,19^ Shelter screening protocols should help women navigate local health services, improve IPV-related health outcomes, speak with the voice of women's trauma, leverage personal strengths, and define next steps for positive screens.

The purpose of this evaluation was to explore the potential effectiveness of TBI screening as a health promotion strategy for shelter-seeking women with IPV head injuries. We wanted to learn if shelter-seeking women, willing to disclose IPV, would accept TBI screening if offered. TBI screening could be considered an effective health promotion strategy for IPV survivors if screening facilitates treatment for positive screens and other unmet health needs.

## Methods

The evaluation was conducted at an urban emergency shelter for women located in the Great Lakes region of the Midwest. All evaluation procedures were presented to the appropriate university social/behavioral sciences Institutional Review Board (IRB) to assure that the recruitment methods, measures, and data aggregation and analyses plan meet IRB certificate criteria for exemption from further oversight.

### Participants

Interested volunteers could participate in an evaluation of their health needs if they were new shelter residents, had one or more head injuries sustained through IPV, and met the inclusion criteria. Intimate partner was defined as any current or former spouse, boyfriend, girlfriend, dating partner, or sexual partner.^1^ Head injury was defined by self-report. The inclusion criteria were (1) completed all shelter intake procedures, (2) age 18 years or older, (3) sustained any head or face injury caused by an intimate partner, (4) speak and read English, and (5) able and willing to use a tablet computer to respond to a secure online survey. The tablet computer and wireless modem were password-protected.

Navigation was restricted to opening, reading, and closing the survey. Clicking submit uploaded the survey responses to a secure campus server, closed the survey, and disconnected the modem. Interested volunteers were not invited to participate if their shelter length-of-stay exceeded 14 days, they only had a few minutes, had privacy concerns, or did not want to use a tablet computer. A designated member of the shelter staff read the approved recruitment flyer at weekly community meetings for residents. The flyer also was posted on announcement bulletin boards in the restricted access living areas of the shelter.

Flyers informed residents of the criteria for participation, survey purpose, number and focus of survey questions, estimated time to complete, and offered one $25 gift card. Flyers also confirmed that participation was voluntary, optional, and not related to any shelter services. Interested volunteers could learn the date, time, and room for the next survey day at the shelter from the designated shelter staff. The survey team had no role in participant recruitment.

### Procedures and measures

The survey is a three-part measure: (1) the HELPS 5-item TBI screen is used to assess head injuries for probability of a related brain injury, (2) questions on perceived health needs assess for health priorities, and (3) demographic characteristics. After participants close the survey, the member of the evaluation team conducting the survey debriefs the participant with an open-ended invitation to comment on their experience. Then participants are given an information sheet that describes the support services available at the shelter and an offer to assist with speaking to shelter staff about obtaining services.

#### HELPS-probability of TBI

The 5-item self-report screening tool is designed to be used by persons without medical training to identify risk of TBI.^20^ A positive screen indicates a probable TBI, meaning the individual needs to have a diagnostic medical evaluation. In other words, the screen is not diagnostic and not a substitute for clinical evaluation. Each item asks about specific events: (1) hits to the head, (2) medical assessment of a head injury, (3) altered consciousness associated with a head injury event, (4) chronic symptoms resulting from head injuries, and (5) any acquired brain injuries from other medical conditions.

Based on the recommendations of Goldin et al.^21^ in their 2016 review of IPV-related TBI screening tools, we added the following IPV prompt: (6) hit in the face, shaken, slammed, pushed, or strangulation. This additional item is recommended because nonfatal strangulation in IPV is common. Strangulation can cause health problems similar to the problems observed with TBI injuries, including neurological changes that can produce central nervous system symptoms, which in IPV survivors may be misdiagnosed as symptoms of mental health problems.^8,11,21,22^ This question was added and the question on acquired brain injury from other medical conditions was removed. Our scores for the 5-item HELPS screen follow the guidelines developed by Picard et al.^20^

A positive HELPS screen for probable TBI means that three conditions are present: (1) the event could have caused a brain injury, (2) the injury was severe (*e.g.*, altered consciousness after the injury, medical attention as a result of the injury), and (3) there now are two or more chronic symptoms that were not present before the injury. If none of the criteria is met, the screen is negative for probable TBI. Loss of consciousness is assessed as a determinate of the extent of brain injuries but, minor TBI might not cause loss of consciousness,^23^ and undetected neurological problems can develop after a head injury without obvious evidence of trauma. In other words, a person can survive IPV and head injuries but sustain a symptomatic but unrecognized TBI.

#### Perceived health needs

Three types of self-reported health needs are assessed: current health needs, unmet health needs, and most important health need today. Health service needs and mental health service needs were assessed with a check-list of common services that are likely to be requested by survivors of IPV with head injuries. Sixteen types of services were identified and defined by reviewing the IPV, TBI, and IPV-related TBI literature.

The framework developed by Pickelsimer et al.^24^ was used to structure a checklist as two questions: have you ever needed the service (yes, no), and have you ever received the service (yes, no). The same paired checklist format is used to assess current health needs (yes, no) and unmet health needs (yes. no). If a health need on a checklist for unmet health need is marked “yes,” the entry opens a corresponding text box question, “What is your most important health need today?” and a final health needs question used to rate the checklist of health needs from 0 (not important at all) to 10 (extremely important). Content analysis methods were used to code and categorize individual needs as medical care, mental health care, dental care, vision care, or other.

#### Demographics

A series of questions with yes or no responses formatted as aggregated categories is used to assess participant's age today, education level, household income, shelter length of stay, current health insurance, current employment, currently parenting a minor age child, race, and ethnicity. There is no additional assessment of gender, genetic, or identity.

### Aggregated analyses

Descriptive statistics were used to characterize the final sample of participants and summarize prevalence rates for categories of HELPS scores, current health needs, unmet health needs, most important health need, general health needs, mental health needs, and important health needs. Scoring used to calculate HELPS score does not distinguish between two clinically distinct conditions: the loss of conscious and alterations of mental status.

If loss of conscious or alteration of mental status, due to IVP-related head injuries, did not occur or cannot be recalled, the response is scored as zero. The scoring equates “no” loss of conscious or alteration of mental status with “cannot recall” without explanatory responses or alternative questions. This means that the proportion of positive TBI screens within the sample could be an underestimation and the proportion of negative TBI screens could include false negatives.

## Results

### Recruitment

Participants were recruited over a period of four consecutive months. During the months of recruitment, the number of new resident intakes ranged from 0 to 3 per week.

### Participants

The final sample of participants (*N* = 18) was educated, primarily black women (68.4%), ages 25 to 34 years (50%), who had been shelter residents for 4 weeks or less (61.1%) (Table 1).

**Table 1.** Participant Characteristics (*N* = 18)

Variable	*n*	%
Age		
18–34	11	61.1
35+	7	38.9
Race/ethnicity^+^		
Black or African American	13	68.4
White	2	10.5
Hispanic/Latina, multiracial	4	21.1
American Indian, Alaska Native, other education		
Some high school	2	11.1
High school diploma	4	22.2
Some college/degree	12	66.7
Household income		
Less than $10,000	12	66.7
$10,000–$29,999	6	33.4
Employment status		
Employed	9	50.0
Unemployed	9	50.0
Health insurance status		
Medicaid/Medicare/Public	12	66.7
Uninsured/no response	6	33.4
Parenting a minor child		
Yes	11	61.1
No	7	38.9
Shelter resident		
<1 week	3	16.7
1–2 weeks	8	44.4
3–4 weeks	3	16.7
>1 month	4	22.2

Race/ethnicity totals exceed 100%.

### HELPS

**FIG. 1.**
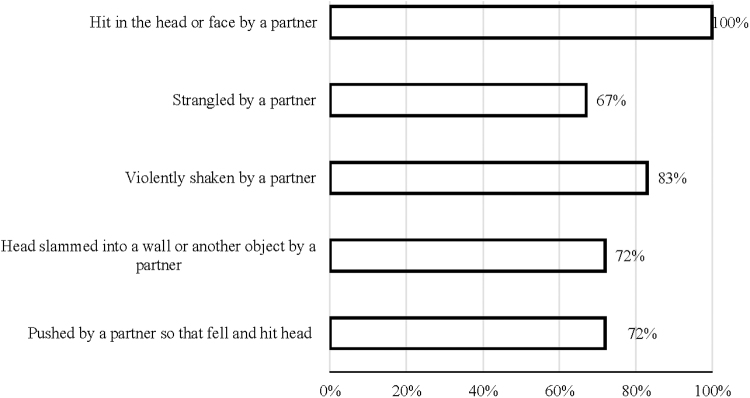
Type of *IPV-related head injury self-reported. Note* Response options: yes, no. IPV, intimate partner violence.

All participants (*N* = 18) self-reported being hit in the head or face by an intimate partner. Most also reported being violently shaken, their head was slammed into a wall or other hard object, and they were pushed so hard that they fell and hit their head (Fig. 1). Two-thirds reported being strangled by an intimate partner. Most (77.8%) had experienced “alteration of consciousness” from an IPV-related head injury. Half (50%) reported loss of consciousness. Most (77.8% had a positive TBI screen). Few (22.2%) reported “no alteration of consciousness.” Half (50%) reported “no loss of conscious.”

### Symptoms experienced daily after IPV-related head injury

Most participants (88.8%) reported at least one daily symptom that they did not have before their IPV head injury. A similar proportion (83.3%) reported three or more daily symptoms and over half (61.1%) reported five or more daily symptoms. Depression (88.9%) was the most common symptom following an IPV head injury, the next three most common symptoms were anxiety (77.8%), difficulty remembering things (72.2%), and headache (66.7%) (Table 2).

**Table 2.** Self-Reported Symptoms Following Intimate Partner Violence-Related Head Injury

Symptom	*N*	%
Headache	12	66.7
Dizziness	11	61.1
Anxiety	14	77.8
Depression	16	88.9
Difficulty remembering things	13	72.2
Difficulty reading or writing	3	16.7
Difficulty solving problems	5	27.8
Difficulty performing work	6	33.3
Changes in relationships	10	55.6
Poor judgment	5	27.8

Totals exceed 100%. Response options: yes, no.

### Health needs

Most (77.8%) participants said their current health care services need was to be seen by a primary care provider but fewer (27.8%) identified primary care as an unmet health need (Table 3). Similar proportions said they needed a support group (72.2%), treatment by a psychiatrist (66.7%), counseling (66.7%), and pain management (61.1%). Two-thirds of the participants reported at least one unmet health need. Also, while participants' health needs showed consistency, their unmet health needs ranged from primary care, brain injury diagnosis, pain management, counseling, and treatment by a psychiatrist, to a support group (Table 3).

**Table 3.** Health Care Service Needs and Unmet Needs

Health care service	Needs		Unmet needs	
	*n*	%	*n*	%
Physical				
Treatment by a primary care provider	14	77.8	5	27.8
Brain injury diagnosis	8	44.4	4	22.2
Treatment for brain injury	5	27.8	1	5.7
Education about brain injuries	6	33.3	2	11.1
Physical therapy	9	50.0	3	16.7
Occupational therapy	7	38.9	3	16.7
Speech therapy	3	16.7	2	11.1
Pain management	11	61.1	5	27.8
Mental				
Behavioral health care	8	44.4	2	11.1
Counseling or therapy	12	66.7	4	22.2
Medication for mental health problems	11	61.1	3	16.7
Treatment by a psychiatrist	12	66.7	5	27.8
Treatment for substance use problems	4	22.2	1	5.7
Support group	13	72.2	5	27.8
Family counseling	8	44.4	2	11.1
Couples counseling	4	22.2	3	16.7

Totals exceed 100%. Response options: yes, no.

More participants described a specific mental health care need (44.4%) as their most important health need (Table 4). Fewer described medical care needs (*e.g.*, asthma, heart problems, hyperthyroidism, headaches, and head pain) as their most important health need today. Most (75%) of the participants who rated the importance of their important health need today said that their need was “extremely important” (mean = 8.7).

**Table 4.** Your Most Important Health Need Today

Category	*n*	%
Medical care	5	27.7
Mental health care	8	44.4
Dental care	2	11.1
Vision care	1	5.7
Other	2	11.1

Response option: select one.

## Discussion

We explored the potential effectiveness of TBI screening as a health promotion strategy for shelter-seeking women with IPV head injuries. The women who volunteered for were well educated black women, parenting a minor age child, and living with extreme poverty. Most of the women had positive (77.8%) screens for probable IPV-related TBI. This finding is consistent with the 75% prevalence rate that Valera and Berenbaum^13^ found for a diverse sample of women with histories of severe IPV, but well exceeds the 33.7% and 50% prevalence rates for black women reported by Cimino et al.,^12^ and Campbell et al.,^11^ respectively.

Most women rated two physical health services (primary care and pain management) and three mental health services (support group, counseling, and treatment by a psychiatrist) as health needs. Less than one-third rated the same services as unmet health needs. Although all participants had sustained an IPV head injury and most experienced alteration of consciousness, most participants had positive TBI screens, and most reported daily symptoms (depression, anxiety, difficulty remembering things, and headache) related to their head injury, yet, few rated “clinical diagnostic services for brain injury” either as a health need or unmet need.

However, the self-reported health needs we found replicate earlier studies of the health concerns of IPV survivors,^25^ adults with positive TBI screens,^26^ and IPV survivors with clinically confirmed TBIs.^27^ Despite their head injury, daily symptoms, and unmet health needs, some participants may not have been aware of their risk of TBI, and the limited scope of our evaluation did not allow distribution of screening results.

Our results also duplicate the findings of cross sectional studies of IPV survivors, with and without self-reported head injuries.^16,28^ Both groups generally report their IPV-related health needs as primary care for pain and mental health care for anxiety and depression. However, it is important to note that IPV can be life threatening. IPV head injuries increase the risk of brain injuries, traumatizing IPV can increase the risk of PTSD with potentially disabling, yet, unrecognized symptoms. Our survey did not ask IPV survivors with head injuries if they were currently or ever had distressing or disabling symptoms of trauma, such as intrusive thoughts, nightmares, hyperarousal, increased sensitivity to internal tension, or avoidant behaviors.^12,15,22,29^

Research shows that IPV survivors with comorbid TBI and PTSD may describe their health needs as physical and psychological distress.^30,31^ Nevertheless, we did not explicitly ask participants if they were traumatized or experiencing any symptoms of PTSD. Shelter-seeking women who have sustained one or more IPV head injuries and screen positive for probable TBI have been traumatized, whether or not they meet clinical diagnostic criteria for PTSD. Common behavioral symptoms of PTSD, such as avoidance and ambivalence, can be missed, yet, may help explain the rate of anxiety and depression in women with IPV head injuries and poor health outcomes in women with untreated TBI.^15,31–35^

The lifetime rate of exposure to IPV, including sexual violence, physical violence, and stalking, for non-Hispanic black women is estimated to be ∼45.1%, the rates of primary care and psychological treatment for IVP are much lower.^36^ Black women can be less likely to seek or report health care for distressing daily symptoms or IPV-related despair^37^ if they are uncertain about the interaction or their care-seeking requires successfully navigating systemic health care barriers and supremacy bias.

For black women, the call to decolonize^38^ health care means eliminating the practice of shaming and blaming black women, the survivors of centuries of sexual, physical, and emotional violence,^39^ with othering behaviors that discredit their pain.^40,41^ Unfortunately, black women with unmet health needs, hoping to avoid distressing health encounters in social spaces where the intersectionality of her race and gender can dehumanize her,^42^ might unsuccessfully seek primary care in hospital emergency departments.^43^

Our results deepen our understanding of the health and healing needs and concerns of urban shelter seeking women with IPV head injuries and positive TBI screens. Wadsworth's et al.^28^ cross sectional findings show that the rate of unmet health needs in women with IPV head injuries (67.7%) is higher than the rate in women who do not have a head injury (53.5%). Women with IPV head injuries may recognize their symptoms and health needs without real awareness of their TBI health risks or the necessity of diagnostic testing for daily headaches from a head injury. They may not have had the opportunity for patient teaching on head injuries severe enough to cause brain injuries. Moreover, until validated TBI risk assessments become available, the individual implications of TBI screening must not be overstated.^11^

In the meantime, the ethical implications of not screening are clear. Diagnostic neurological testing, if available, can be costly and, for some, parenting and employment circumstances could make testing feel like a risk without a benefit.^8^ Emergency shelters that could offer basic screening for TBI and unmet health needs can be a practical “next step” alternative. Shelters seek to create a safe space for women to make choices and make plans. Screening, as a routine shelter protocol for women with IPV head injuries, could give women the information and support they need to obtain a clinical diagnosis of their injury by increasing awareness and decreasing anxious avoidance as barriers to help seeking.^44^

Our sample frame and size limit any generalization of our results to similar populations. As a preliminary first step, we wanted to know if shelter seeking women, willing to disclose IPV-related head injuries, would accept TBI screening if offered. Despite the self-selection bias in our recruitment frame and small sample, our participants were representative of women with IPV head injuries and positive TBI screens. However, psychometric confirmation of the measurement validity and reliability of the widely used HELPS is needed.^21^

Despite the problem of using zero to score no loss of consciousness or alteration of conscious and cannot recall and the physiological differences between a head injury and strangulation, HELPS scores indicate probability for TBI appear to be consistent in diverse populations. These scoring limitations can mean that our rate of positive TBI screens might be an underestimation and our rate of negative TBI screens might include false negatives.

Future studies designed to explore alternative recall questions are needed (*e.g.*, Did you ever feel like you had just woke-up?). Our results also are subject to the inherent limits of self-selection bias in the women willing to take the survey and retrospective self-reports of a violent injury. Finally, although all participants met shelter criteria to become residents and our rate of positive TBI screens is consistent with the IPV literature, without clinical diagnosis or adjusting for PTSD symptoms as confounding factors, our results are a preliminary assessment of screening shelter-seeking women with IPV head injuries for TBI and unmet health needs.

## Conclusion

TBI screening could be considered an effective health promotion strategy for IPV survivors if screening facilitates treatment for positive screens and other unmet health needs.^18,45–47^ Further research is needed to properly assess this.

## Abbreviations Used

IPV: intimate partner violence
PTSD: posttraumatic stress disorder
TBI: traumatic brain injury
